# GASS-tly side effects: antipsychotic monitoring for inpatients across NHS Lanarkshire

**DOI:** 10.1192/bjo.2021.293

**Published:** 2021-06-18

**Authors:** Cristina Paton, Siggi Hammond, James Hills, Leah Jones, Eugene Wong

**Affiliations:** 1 University Hospital Hairmyres; 2 University Hospital Wishaw

## Abstract

**Aims:**

Best practice in the prescribing of antipsychotic therapy includes monitoring for medication side effects. National guideline SIGN 131 advises the use of a validated side effect scale, for example the Glasgow Antipsychotic Side-effect Scale (GASS). Local recommendation in NHS Lanarkshire advises that patients prescribed antipsychotic therapy should be offered GASS at each contact and after initiation or titration. We aimed to improve compliance with antipsychotic side effect monitoring for inpatients in general adult psychiatry across two hospital sites in NHS Lanarkshire.

**Method:**

We conducted a full-cycle audit. In October 2020, we took a cross-sectional sample of inpatients in general adult psychiatry in University Hospital Hairmyres and University Hospital Wishaw who were prescribed antipsychotic therapy for a functional psychotic disorder. For these inpatients, if applicable, we identified whether GASS had been completed on admission (OA), whether GASS had been completed after initiation or titration of antipsychotic therapy (I/T), and whether GASS had been acknowledged and discussed at consultant-led multi-disciplinary team meeting (MDT). Thereafter, we implemented several targeted interventions in order to improve compliance. In January 2021, we completed the cycle by taking a new cross-sectional sample of inpatients fulfilling identical parameters.

**Result:**

First cycle in October 2020 (n = 27) showed compliance OA of 4.2%, I/T of 9.5%, and MDT of 3.7%. Our interventions included a presentation at trust-wide clinical governance meeting; a presentation at one of the weekly departmental teaching sessions in psychiatry; an email summarising the audit to consultants in general adult psychiatry; meetings with senior charge nurses for each ward; and inclusion of GASS as part of routine admission paperwork. Re-audit in January 2021 (n = 23) showed compliance OA of 11.1%, I/T of 40.0%, and MDT of 21.7%.

**Conclusion:**

Our full-cycle audit led to modest improvement in documented monitoring for antipsychotic side effects. There was relatively greater improvement in prescriber-led outcomes I/T and MDT, suggesting increased prescriber awareness. However, rather than reliance on individual prescribers to ensure compliance, consideration of GASS alongside monitoring of other physical health parameters would likely result in greater and more sustained improvement. In NHS Lanarkshire there is ongoing work to this end, ultimately with the intention to set up a defined antipsychotic physical health monitoring schedule, integrated across inpatient and community care.

